# Variation in hybridogenetic hybrid emergence between populations of water frogs from the *Pelophylax esculentus* complex

**DOI:** 10.1371/journal.pone.0224759

**Published:** 2019-11-01

**Authors:** Dmitrij Dedukh, Julia Litvinchuk, Anton Svinin, Spartak Litvinchuk, Juriy Rosanov, Alla Krasikova

**Affiliations:** 1 Saint-Petersburg State University, Saint-Petersburg, Russia; 2 Mari State University, Mari El Republic, Russia; 3 Institute of Cytology, Russian Academy of Sciences, Saint-Petersburg, Russia; 4 Department of Zoology and Physiology, Dagestan State University, Makhachkala, Russia; Virginia Tech Virginia, UNITED STATES

## Abstract

Many closely related species are capable of mating to produce hybrid offspring, which are usually sterile. Nevertheless, altering the gametogenesis of hybrid offspring can rescue hybrids from sterility by enabling asexual reproduction. Hybridogenesis is one of the most complicated asexual reproductive modes, and it includes drastic genome reorganization only in the germline; this is achieved through elimination of one parental genome and duplication of the remaining one to restore diploid chromosomal set and overcome blocks in meiotic progression. We investigated a model of hybridogenesis, namely, water frogs from the *Pelophylax esculentus* complex, for the emergence of asexual reproduction. Further, we assessed the impact of its asexual reproduction on the maintenance of interspecies hybrids from two populations on the western edge of the *P*. *esculentus* range, in which hybrids coexist with either both parental species or with only one parental species. After analysing tadpole karyotypes, we conclude that in both studied populations, the majority of diploid hybrid males produced haploid gametes with the *P*. *ridibundus* genome after elimination of the *P*. *lessonae* genome. Hybrid females exhibited problems with genome elimination and duplication; they usually produced oocytes with univalents, but there were observations of individual oocytes with 13 bivalents and even 26 bivalents. In some hybrid tadpoles, especially F1 crosses, we observed failed germ cell development, while in tadpoles from backcrosses, germ cells were normally distributed and contained micronuclei. By identifying chromosomes present in micronuclei, we estimated that the majority of tadpoles from all crosses were able to selectively eliminate the *P*. *lessonae* chromosomes. According to our results, hybridogenesis in hybrids can appear both from crosses of parental species and crosses between sexual species with hybrid individuals. The ability to eliminate a genome and perform endoreplication to ensure gamete formation differed between male and female hybrids from the studied populations. Some diploid hybrid females can rarely produce not only haploid gametes but also diploid gametes, which is a crucial step in the formation of triploid hybrids.

## Introduction

Biological species are separated from each other via different prezygotic and postzygotic barriers, which are frequently disrupted, resulting in the emergence of hybrids [1,2]. Hybrids are generally sterile because of the disruption of gametogenesis and meiosis, leading to aberrant gamete formation [2,3]. To overcome chromosome pairing problems, some animal hybrids exhibit sophisticated gametogenetic pathways, where meiosis in particular is modified to produce gametes without recombination [4–6]. These evolutionary novelties give rise to a great variety of independently emerged clonal and hemiclonal organisms, such as parthenogenesis (occurring in some fishes, reptiles), gynogenesis (occurring in various fishes), kleptogenesis (amphibians) and hybridogenesis (fishes, amphibians) [4,7]. Although many examples of asexual hybrids are known, the cellular pathways of achieving clonal reproduction *de novo* and the consequences of newly formed clonal hybrids remain unknown [5,6].

One of the most intriguing asexual reproductive modes, hybridogenesis, was found in hybrids from the European water frog complex (*Pelophylax esculentus*) [8]. Hybridogenetic frogs, *Pelophylax esculentus* (RL), appear after crosses of two parental species: the marsh frog *P*. *ridibundus* (RR) and the pool frog *P*. *lessonae* (LL) [9]. During hybridogenetic reproduction, one of the parental genomes is eliminated from the gonial cells of diploid hybrids, while the other undergoes endoreplication and is transmitted to gametes without recombination [8,10,11]. These modifications ensure hybrid fertility but require regular backcrosses with one of the parental species to restore the hybrid chromosomal set [8]. Triploid hybrids can substitute parental species to retain the ability of recombination, but they still depend on diploid hybrids for reproduction [12–14]. Throughout the range of the European water frog, populations with various combinations of hybrids and sexual species were observed: 1) both parental species without the formation of hybrids (R-L); 2) both parental species with diploid hybrids (R-L-E); 3) diploid or triploid hybrids coexisting with one parental species (L-E and R-E); 4) diploid and triploid (rarely, tetraploid, which did not reach maturity) hybrids coexisting with one parental species (L-E-t and R-E-t); and 5) diploid and triploid (sometimes, tetraploid) hybrids occurring in the absence of both parental species (E-t) [15–17]. It was suggested that in such population systems, hybrids gradually switch from those that depend on parents to emerge (R-L-E systems) and reproduce (L-E and R-E systems) to independent on parents evolutionary units (E systems) [18–21].

Hybrids have a broad range from France in the west to Volga River in the east, but their distribution is uneven [15–17]. Hybrids are widespread in Central Europe, but they are unexpectedly rare in the eastern and southern ranges of the parental species [16,17, 22–24]. Despite hybridization occurring from both parental species, in the western part of the range, *P*. *esculentus* is usually absent or sterile; this result is probably caused by the inability of *P*. *esculentus* to reproduce hybridogenetically [16,17,22]. Only in some restricted localities in the eastern range of a parental species, *P*. *esculentus* is known to be abundant [17,22–24]. Corresponding populations were found in the Mari El Republic of Russia, where hybrids coexisted either with both parental species (the R-L-E system) or with only *P*. *lessonae* (the L-E system) [22,25–27]. Most European L-E systems that have been investigated are characterized by a stable genetic structure with a high proportion of hybrids, which often contain both diploids and triploids [15,16,28]. Male and female diploid hybrid frogs in such systems typically eliminate the *P*. *lessonae* genome, transmitting the *P*. *ridibundus* genome to gametes [15,16,28–30]. However, taking into account that the studied L-E systems are separated from central European systems and are located almost on the western border of the *P*. *lessonae* range [17,22], we first asked whether male and female hybrids are able to eliminate the *P*. *lessonae* genome and produce gametes with the *P*. *ridibundus* genome.

R-L-E systems are considered either recent or unstable [16,31]; thus, understanding the mechanisms of hybrid reproduction in such systems can shed light on the emergence of hybridogenesis itself during the initial stages of speciation via interspecies hybridization. It has been suggested that hybrids can occur from primary crosses of both parental species or after regular crosses with one of the parental species, assuming the ability of such hybrids to reproduce by hybridogenesis [15,16,31]. However, the ability of hybrids to reproduce in such systems is not well known [15–17,31,32]. Herein, we aimed to determine whether *P*. *esculentus* males and females from R-L-E systems can reproduce hybridogenetically or if they appear only after primary crosses with parental species.

In a series of studies, genome elimination in hybridogenetic water frogs was shown to occur premeiotically during the early embryonic development of tadpoles [14,33,34]. In particular, it was demonstrated that chromosomes from one of the parental genomes were eliminated by micronuclei formation, and they were subsequently degraded [14,33,34]. In the present study, we aimed to define the morphology of hybrid tadpole gonads, assess the presence of germ cells and discover which genome is present in the micronuclei. In particular, we asked whether micronuclei formation and selectivity in elimination of the *P*. *lessonae* genome differs between F1 hybrid tadpoles and hybrid tadpoles from backcrosses.

To investigate hybrid reproduction in L-E and R-L-E systems from the Mari El Republic, we identified the karyotypes of tadpoles obtained from laboratory crosses of parental species and hybrids. Furthermore, we carried out cytological analysis of the chromosomal sets transmitted in the oocytes of hybrid females. This allowed us to suggest roles for hybrid males and females in the formation of gametes and progeny. To determine whether genome elimination occurs in the gonads of hybrid tadpoles from different crosses, we also performed morphological analysis of gonads and identified the genome transmitted to the micronuclei.

## Materials and methods

### Animal capture

Water frogs from the *P*. *esculentus* complex were captured from 9 locations, and they included two pure populations of parental species (R system for *P*. *ridibundus* and L system for *P*. *lessonae*), 3 L-E systems and 4 R-L-E systems. They were captured in the Mari El Republic of Russia during May 2015, May 2016, and May 2017. We collected 91 frogs of both sexes, of which 39 individuals were hybrids; 12 were *P*. *ridibundus* and 40 were *P*. *lessonae* (**S1 Table**). All manipulations with animals were carried out in accordance with national and international guidelines. This study did not involve endangered or protected species. All specimens were collected outside of protected areas within Russia; thus, no specific permissions were required. Techniques used in the capture, breeding, tissue sampling and euthanasia sought to minimize animal suffering. Before euthanasia, each individual was anaesthetized by submersion in a 1% solution of 3-aminobenzoic acid ethyl ester (MS 222). All procedures were approved by the Local Animal Ethics Committee of Saint-Petersburg State University (# 131-03-3).

### Species identification

Primary identification of all three species of the *P*. *esculentus* complex was performed using visual analysis of morphological features such as colour pattern, size and shape of inner metatarsal tubercle and colour of resonators of males [16,35]. To confirm the genome composition and ploidy of captured animals, we measured the DNA content per nucleus using a flow fluorimeter at the Institute of Cytology, Russian Academy of Sciences, St. Petersburg according to previously published protocols [36,37]. Additionally, karyotype analysis was performed using fluorescent *in situ* hybridization (FISH) with telomeric and centromeric probes (detailed description of the method is presented below and in [38]).

### Crossing experiments

After the species genome composition and sex of each individual were determined, we conducted laboratory crosses of parental species with each other and hybrid animals from L-E and R-L-E systems according to [14]. Tadpoles were reared through stage 28 and beyond according to Gosner [39], and then they were randomly selected for further analysis. Tadpoles were placed in an anaesthetic solution, and gills, intestines and tails were dissected and fixed in ethanol and glacial acetic acid (in the ratio 3:1) to enable visualization of metaphase chromosomes. In addition, gonads of tadpoles were dissected and fixed in 1% paraformaldehyde solution for 90 min. After fixation, gonads were placed in 1×PBS with 0.02% NaN_3_ for long-term storage.

### Preparation of lampbrush and metaphase chromosomes

To obtain metaphase chromosomal spreads, fixed gills were placed in 150 μl of 70% acetic acid and were disintegrated using two forceps. The resulting cell suspension was dropped onto slides that were preheated to 60°C. After the solution evaporated, metaphase spreads and interphase nuclei remained on the slide.

After crossing experiments, the growing oocytes from hybrid females were used for preparation of lampbrush chromosomes [38]. After euthanasia, ovaries were removed. The growing oocytes were separated from the ovarian fragments in OR2 solution (82.5 mM NaCl, 2.5 mM KCl, 1 mM MgCl_2_, 1 mM CaCl_2_, 1 mM Na_2_HPO_4_, 5 mM HEPES (4-(2-hydroxyethyl)-1-piperazineethanesulfonic acid); pH 7.4). Then, the oocytes were placed in a 5:1 medium (83 mM KCl, 17 mM NaCl, 6.5 mM Na_2_HPO_4_, 3.5 mM KH_2_PO_4_, 1 mM MgCl_2_, 1 mM DTT (dithiothreitol); pH 7.0–7.2) for isolation. The membrane of the oocyte was torn using two forceps, and nuclei were extracted. After being placed in one-fourth strength 5:1 medium containing 0.1% paraformaldehyde and 0.01% 1 M MgCl_2_, nuclear membranes were gently removed using forceps and thin tungsten needles. Microsurgical manipulations were carried out using a Leica MZ16 stereomicroscope (Leica-Microsystems).

### Fluorescent *in situ* hybridization

Fluorescent *in situ* hybridization (FISH) was performed on metaphase and lampbrush chromosomes. The slides containing metaphase chromosomes were treated with RNase A (100–200 μg/ml) for one hour, and pepsin (0.005%, diluted in 0.01 N HCl) for 8 min. Two kinds of probes were used for FISH: (1) a single-stranded oligonucleotide telomeric probe (TTAGGG)_5_ conjugated with biotin or fluorochrome Cy3, (2) a biotin labelled probe, which was obtained from the genomic DNA of *P*. *ridibundus* by PCR with the following primers for the RrS1 centromeric repeat (according to [40]):

5`-AAGCCGATTTTAGACAAGATTGC-3`;

5`-GGCCTTTGGTTACCAAATGC-3`.

In the case of the oligonucleotide probe, the hybridization mixture contained 40% formamide, 10% dextran sulfate, 2×SSC, 5 ng/μl labelled probe and a 10–50-fold excess of tRNA. In the case of the long centromeric probe, the hybridization mixture contained 50% formamide, 10% dextran sulfate, 2×SSC, 5 ng/μl labelled probe and a 10–50-fold excess of salmon sperm DNA. After application of the hybridization mixture, slides were covered with cover slips, sealed with rubber glue and denatured at 75°C for five min. Slides were incubated for 12–24 hours at room temperature (RT) in the case of the oligonucleotide probe and at 37°C in the case of the long centromeric probe. After hybridization, slides with the oligonucleotide probe were washed in 2×SSC at 42°C, while slides with the centromeric probe were washed in 0.2×SSC at 60°C. Biotin was detected by avidin conjugated to fluorochrome Cy3 (Jackson ImmunoResearch Laboratories). Afterward, slides were sequentially treated with an ethanol series (50%, 70% and 96%), dried, and mounted in DABCO (Merck) antifade solution containing 1 μg/ml DAPI.

### Whole-mount fluorescent *in situ* hybridization

For 3D-FISH, gonads dissected from tadpoles of various developmental stages were used. The probe for the centromeric repeat used in hybridization was made as described above. Before hybridization, tissues were permeabilized by treatment with 1% Triton X-100 made in 1×PBS at RT for 4–5 hours. After washing in 1×PBS for 10 min, the tissues were pretreated with 40% formamide, 10% dextran sulfate, and 2×SSC for 3–4 hours at 37°C. After the pretreatment solution was removed, the hybridization mixture (50% formamide, 10% dextran sulfate, 2×SSC, 5 ng/μl labelled probe and 10–50-fold excess of salmon sperm DNA) was added. Denaturation was performed at 82°C for 15 min, and then the tissue was incubated for 24 hours at RT. Afterwards, tissues were washed in 0.2×SSC at 42°C. Biotin was detected using avidin conjugated to fluorochrome Cy3 (Jackson ImmunoResearch Laboratories). Tissues were stained using DAPI diluted in 1×PBS (1 μg/ml) for 12–24 hours. Before observation under a confocal microscope, tissues were placed on coverslips with a drop of DAPI (1 μg/ml) (Sigma) in antifade solution (DABCO, Merck).

### Fluorescent microscopy and laser scanning confocal microscopy

Slides with chromosome spreads were analysed using a fluorescence microscope, Leica DM 4000, equipped with a monochrome digital camera DFC350 FX and filter cubes corresponding to fluorochromes Alexa488, Cy3, and DAPI (Leica-Microsystems). The LAS X core computer program was used to obtain and process colour images.

Analysis of the 3D morphology of the gonads and whole-mount FISH was performed by laser scanning confocal microscopy (using a Leica TCS SP5 based on the inverted microscope Leica DMI 6000 CS, Leica-Microsystems). Nuclei were scanned in XYZ planes using lens HC PL APO 40×. Images were obtained by the LAS AF program (Leica-Microsystems, Germany).

## Results

### Crosses of diploid hybrids with *P*. *lessonae* and both parental species produce viable diploid hybrid tadpoles

**Fig 1:**
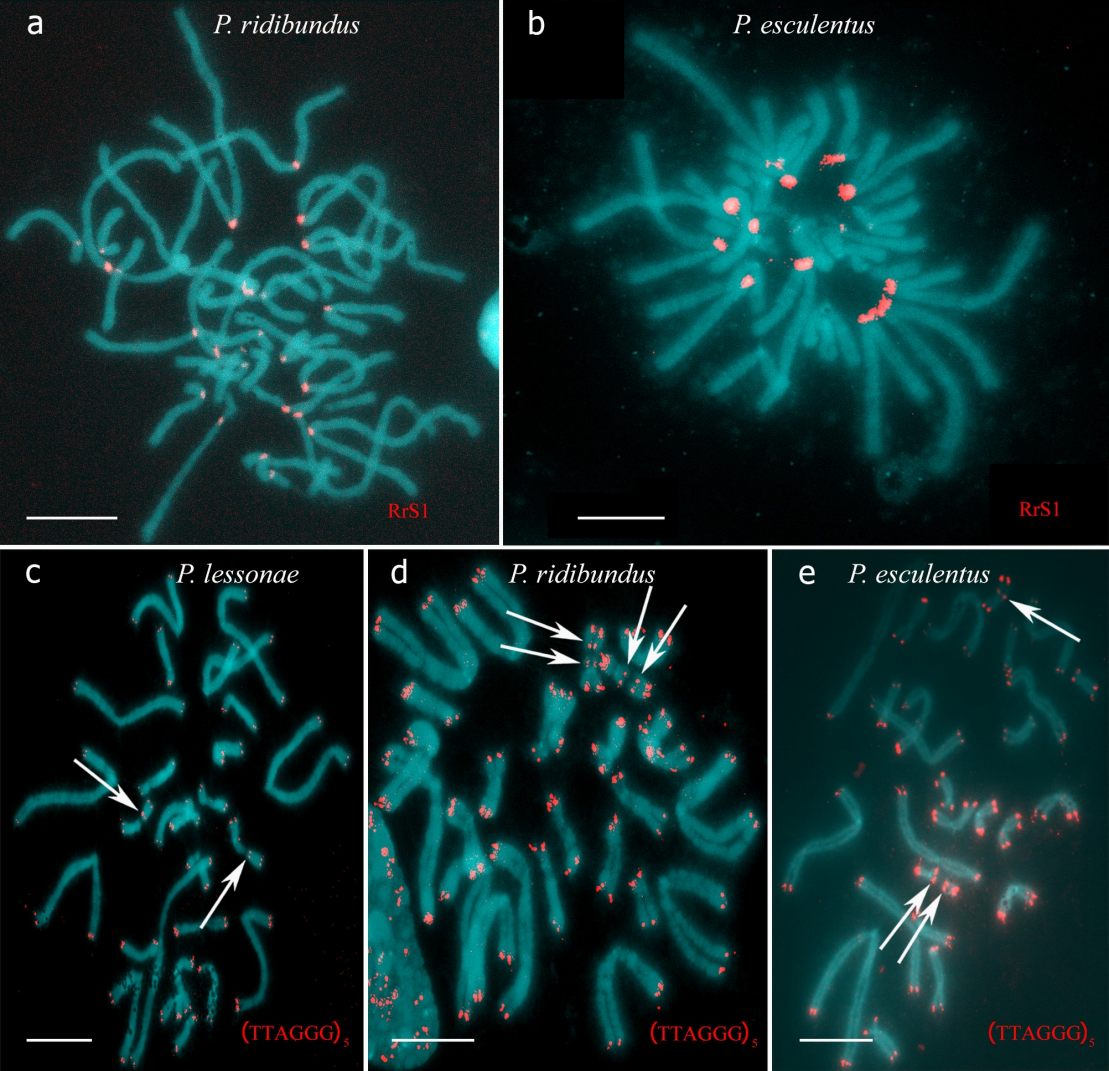
Water frog karyotype identification using FISH with species-specific markers to centromeric repeat (RrS1) and interstitial (TTAGGG)_n_ repeat sites (ITSs). Metaphase chromosomes after FISH using the centromeric probe (RrS1) (a, b), which detects only *P*. *ridibundus* but not *P*. *lessonae* centromeres (a, b). Metaphase chromosomes after FISH using the probe to (TTAGGG)_n_ repeat, which distinguishes one or two ITSs (arrows) in parental NOR-bearing chromosomes (c-e). Parental species were identified as *P*. *ridibundus* (a, d) and *P*. *lessonae* (c), while tadpoles were identified as diploid hybrids (b, e). Scale bars = 10 μm.

To evaluate the hybrid contribution to the formation of viable progeny, we performed 29 artificial crosses of diploid hybrid males and females from the studied L-E and R-L-E systems with one of the parental species **(Table 1)**. Tadpoles were obtained from 8 crosses of hybrid males from L-E systems and 2 crosses of hybrid males from R-L-E systems. Another 19 attempts were unsuccessful either because eggs were unfertilized or because females did not spawn **(S1 Table)**. Notably, no hybrid females produced progeny in any of our crossbreeding experiments. Identification of tadpole karyotypes revealed only diploid hybrid tadpoles in crosses of *P*. *lessonae* with diploid hybrid males from either the L-E or R-L-E systems **(Table 1, Fig 1)**. Thus, our results indicated that these males produced sperm with the *P*. *ridibundus* genome and eliminated the *P*. *lessonae* genome.

**Table 1:** Results of crossing *P*. *ridibundus* with *P*. *lessonae* (marked orange), *P*. *lessonae* individuals with each other (marked red) and diploid hybrids from L-E (marked blue) and R-L-E (marked green) systems.

Female	Population system	Male	Population system	Crosses id	Number of analyzed tadpoles	Tadpoles
*P*. *ridibundus*	R-L-E system	*P*. *lessonae*	R-L-E system	6_2015	17	*P*. *esculentus*
		*P*. *lessonae*	R-L-E system			
*P*. *ridibundus*	R-L-E system	*P*. *lessonae*	R-L-E system	16_2015	2	*P*. *esculentus*
*P*. *ridibundus*	R-L-E system	*P*. *lessonae*	L-E system	11_2016	13	*P*. *esculentus*
		*P*. *lessonae*	L-E system			
*P*. *ridibundus*	R-L-E system	*P*. *lessonae*	L-E system	13_2016	16	*P*. *esculentus*
		*P*. *lessonae*	L-E system			
*P*. *ridibundus*	R system	*P*. *lessonae*	L system	1_2017	7	*P*. *esculentus*
*P*. *lessonae*	L-E system	*P*. *esculentus*	L-E system	5_2016	3	*P*. *esculentus*
*P*. *lessonae*	R-L-E system	*P*. *esculentus*	L-E system	6_2016	12	*P*. *esculentus*
*P*. *lessonae*	L-E system	*P*. *esculentus*	L-E system	11_2015	11	*P*. *esculentus*
*P*. *lessonae*	L-E system	*P*. *esculentus*	L-E system	12_2015	7	*P*. *esculentus*
*P*. *lessonae*	L-E system	*P*. *esculentus*	L-E system	13_2015	23	*P*. *esculentus*
*P*. *lessonae*	L-E system	*P*. *esculentus*	L-E system	14_2015	16	*P*. *esculentus*
*P*. *lessonae*	L-E system	*P*. *esculentus*	L-E system	26_2015	9	*P*. *esculentus*
*P*. *lessonae*	L-E system	*P*. *esculentus*	L-E system	27_2015	19	*P*. *esculentus*
*P*. *lessonae*	L-E system	*P*. *esculentus*	R-L-E system	19_2015	28	*P*. *esculentus*
*P*. *lessonae*	R-L-E system	*P*. *esculentus*	R-L-E system	30_2015	20	*P*. *esculentus*
*P*. *lessonae*	R-L-E system					
*P*. *lessonae*	L system	*P*. *lessonae*	L system	1_2017	3	*P*. *lessonae*

Additionally, we produced 8 crosses of both parental species **(Table 1)**. We obtained progeny from 4 crosses of *P*. *ridibundus* females with *P*. *lessonae* males and from 1 cross of *P*. *lessonae* females and *P*. *ridibundus* males (**Table 1, Fig 1**). As a control, we also crossed *P*. *lessonae* individuals, which resulted in progeny (**Table 1)**.

We conclude that in the studied L-E systems, crosses of *P*. *lessonae* females with *P*. *esculentus* males give rise to hybrid progeny. In the studied R-L-E systems, hybrid progeny can appear after crosses of *P*. *lessonae* females with diploid hybrid males and after primary crosses of the two parental species.

### Hybrid females from studied populations exhibit variability in oocyte genome composition

Ovaries from hybrid females were undeveloped and contained oocytes corresponding to I-III stages of development according to Dumont [41]. We analysed the genome composition of oocytes from 6 diploid hybrid females from two different L-E systems and 6 diploid hybrid females from 4 different R-L-E systems (**Table 2**). To confirm the results from the morphological analysis, we performed FISH with either (TTAGGG)_5_ or centromeric RrS1 repeat probes.

**Table 2:** Composition of diplotene oocytes from diploid hybrid frogs from L-E (marked blue) and R-L-E (marked green) systems.

Genotype	Frog`s id	Population system	Locality	Composition of oocytes		
				13 bivalents	26 bivalents	26 univalents
*P*. *esculentus*	1K_2017_f	L-E system	Kuguvan	6		1
*P*. *esculentus*	2K_2017_f	L-E system	Kuguvan			19
*P*. *esculentus*	3K_2017_f	L-E system	Kuguvan	28		15
*P*. *esculentus*	4K_2017_f	L-E system	Kuguvan		1	9
*P*. *esculentus*	5K_2017_f	L-E system	Kuguvan	6		
*P*. esculentus*	7_2015_f	L-E system	Oshlamoochash			18
*P*. *esculentus*	23_2015_f	R-L-E system	Krasnooktyabr'skiy			11
*P*. *esculentus*	9_2015_f	R-L-E system	Krasnooktyabr'skiy	23		
*P*. *esculentus*	1N_2017_f	R-L-E system	Nol'ka	1	2	24
*P*. *esculentus*	1M_2017_f	R-L-E system	Medvedevo	16		2
*P*. *esculentus*	5_2014_f	R-L-E system	Chermyshevo		2	16
*P*. *esculentus*	2_2014_f	R-L-E system	Chermyshevo			16

* hermaphroditic individual that had both testes and an ovary

**Fig 2:**
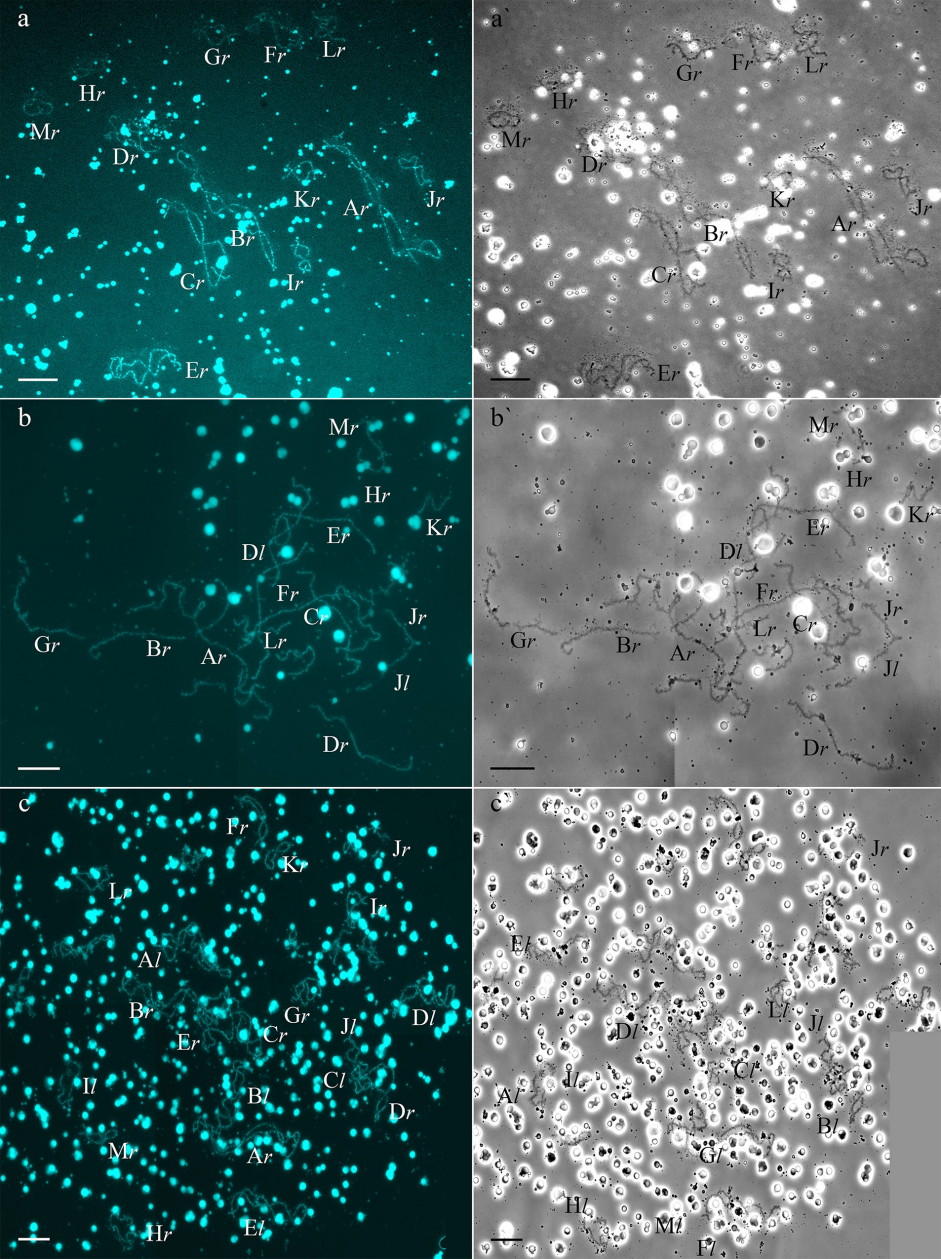
High variability of lampbrush chromosome karyotypes from oocytes of diploid hybrid frogs. Lampbrush chromosome sets from oocytes of diploid hybrid frogs included 13 *P*. *ridibundus* bivalents (a, a`), 26 univalents (b, b`) and 26 bivalents (c, c`). Lampbrush chromosomal sets with 26 bivalents and 26 univalents include 13 bi- or univalents that correspond to *P*. *ridibundus* chromosomes, while another 13 bi- univalents correspond to *P*. *lessonae* chromosomes. Lampbrush chromosomes are numbered alphabetically; italic type indicates correspondence of the identified chromosome to genotype of parental species: *l*–to *P*. *lessonae*, *r*–to *P*. *ridibundus*. Chromosomes were counterstained with DAPI (a, b, c). Corresponding phase-contrast micrographs are shown (a`, b`, c`). Scale bars = 50 μm.

Detailed analysis of 85 oocytes from 5 hybrid females from L-E systems, revealed extremely variable genome composition. One female formed oocytes containing 13 bivalents of *P*. *ridibundus* (**Table 2)**. Another female produced oocytes with only 26 univalents, corresponding to the *P*. *ridibundus* and *P*. *lessonae* chromosomes (**Table 2, Fig 2b and 2b`, S1A Fig**). Two females formed oocytes containing both the 26 univalents corresponding to *P*. *ridibundus* and *P*. *lessonae* chromosomes and the 13 bivalents corresponding to *P*. *ridibundus* chromosomes (**Table 2, Fig 2a and 2a`**). Moreover, in one hybrid female, we found oocytes with both 26 univalents and 26 bivalents (**Table 2, Fig 2c and 2c`, S1B Fig**), where 13 uni- or bivalents corresponded to *P*. *ridibundus* chromosomes, and 13 uni- or bivalents corresponded to *P*. *lessonae* chromosomes. Thus, during gametogenesis of these females, genome elimination and endoreplication occurred only in some oogonial cells; in the others, genome elimination and endoreplication were absent, leading to high variation in oocyte genome composition.

In addition, in the L-E system, we found one hermaphroditic individual that had both testes and an ovary. All 18 oocytes observed in this animal included 26 univalents; 13 chromosomes corresponded to *P*. *ridibundus* chromosomes and 13 chromosomes corresponded to *P*. *lessonae* chromosomes. Such oocytes with univalents indicate the absence of genome elimination and duplication (**Table 2, marked by an asterisk**).

After detailed analysis of more than 110 oocytes from 6 hybrid females from 4 different populations of R-L-E systems, we also observed variable genome compositions (**Table 2**). In two hybrid females, we observed only oocytes with 26 univalents, corresponding to *P*. *ridibundus* and *P*. *lessonae* chromosomes (**Table 2**). Another female produced only oocytes with 13 bivalents corresponding to *P*. *ridibundus* chromosomes (**Table 2**). Another produced oocytes with 26 univalents, which represented the chromosomes of both parental species, in addition to 13 bivalents corresponding to the *P*. *ridibundus* chromosomes (**Table 2**). Moreover, one female produced oocytes with 13 bivalents corresponding to *P*. *ridibundus* chromosomes as well as 26 univalents and 26 bivalents, where 13 uni- or bivalents correspond to *P*. *ridibundus* chromosomes, and 13 uni- or bivalents correspond to *P*. *lessonae* chromosomes (**Table 2**). Our results suggest that in R-L-E systems, some hybrid females are able to eliminate and duplicate genomes during gametogenesis; however, aberrations in these processes seem to be more frequent than they are in hybrid females from L-E systems.

### Aberration of early gametogenesis in hybrid tadpoles

**Fig 3:**
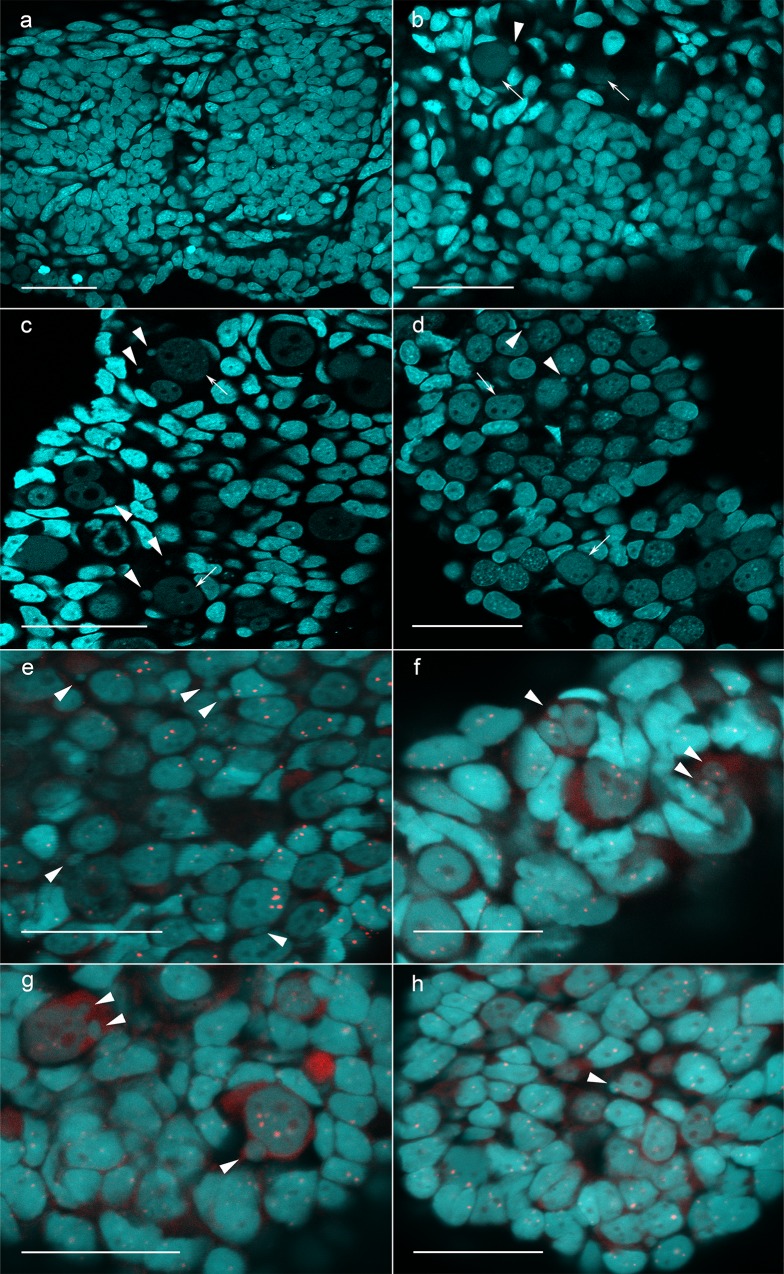
Germ cells and micronuclei in gonads of hybrid tadpoles. Observation of germ cells and micronuclei in the gonads of F1 hybrids (a, c) and hybrids obtained after crossing *P*. *lessonae* females and hybrid males (b, d). Examples of various distribution of germ cells (a-d) from their complete absence (a) to high number of germ cells (c, d). Identification of *P*. *ridibundus* chromosomes incorporated in the micronuclei (e-h) using FISH with the centromeric RrS1 repeat probe specific for *P*. *ridibundus* centromeres in F1 hybrids (e), hybrids produced from crosses of *P*. *lessonae* females and hybrid males from R-L-E system (f) and hybrids produced from crosses of *P*. *lessonae* females and hybrid males from L-E system (g, h). Arrows indicate germ cells, arrowheads indicate micronuclei. Scale bars = 50 μm.

**Fig 4:**
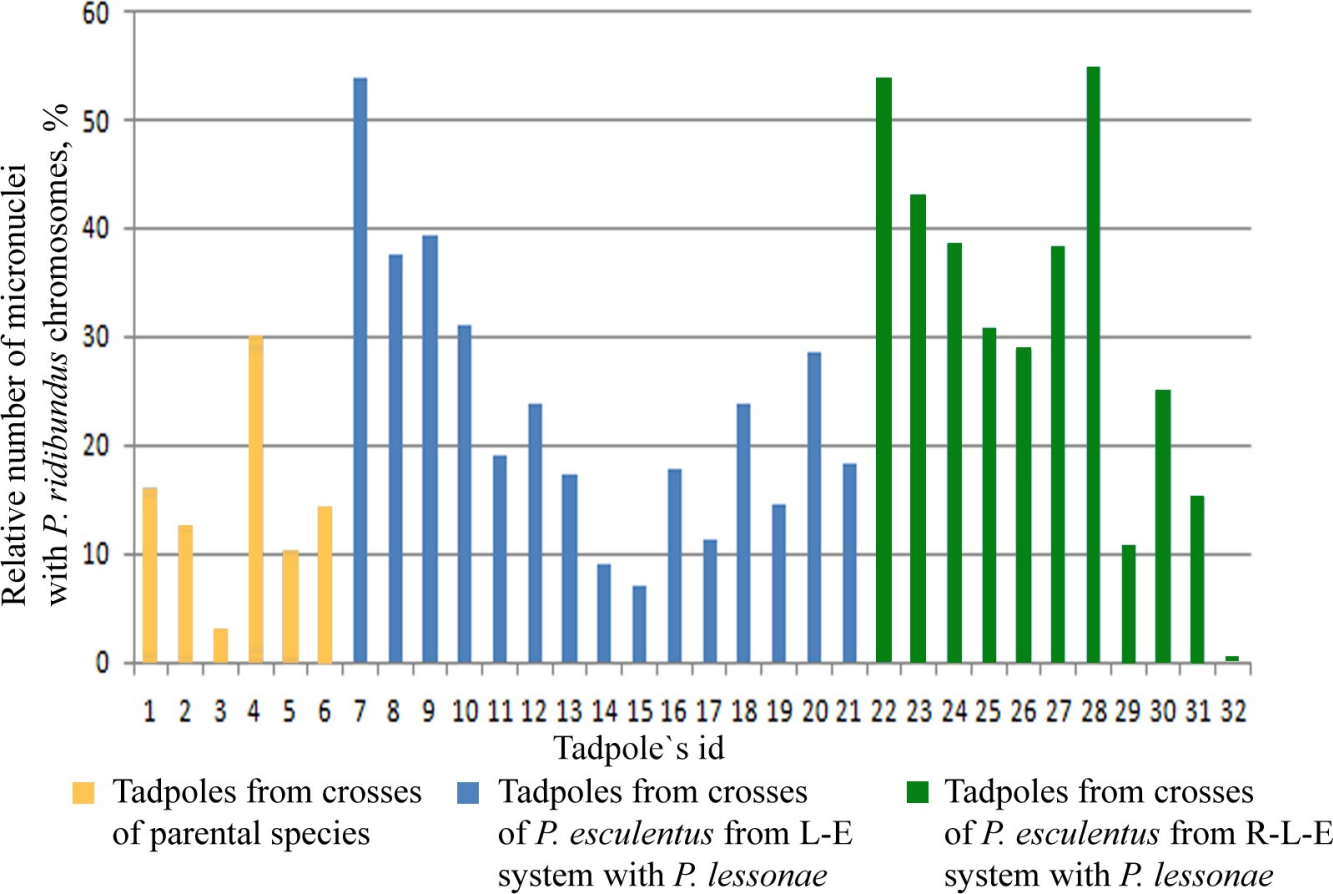
Relative number of micronuclei bearing *P*. *ridibundus* chromosomes in hybrids obtained from various crosses.

To verify the potential fertility of tadpoles from different crosses, we examined the gonads of tadpoles from hybrids and both parental species during stages of development 29–36 according to Gosner [39], which we analysed using laser scanning confocal microscopy. Of the 41 diploid hybrid tadpoles from 6 different crosses of *P*. *lessonae* females and *P*. *esculentus* males from L-E systems, 2 tadpoles did not have germ cells, and 9 had a lower number of germ cells than were observed in *P*. *lessonae* tadpoles (**Fig 3A and 3B, S2 Table**). In 30 other hybrid tadpoles, the number of germ cells was similar to the number of germ cells in *P*. *lessonae* tadpoles (**Fig 3C and 3D, S2 Table**). Notably, in 36 tadpoles, we were able to detect DAPI-positive micronuclei in the cytoplasm of germ cells. To determine whether these micronuclei include *P*. *lessonae* or *P*. *ridibundus* chromosomes, we performed 3D-FISH with a probe specific to centromeric RrS1 repeat on 15 tadpoles from 5 different crosses (**Fig 3E–3H**). In tadpoles from 5 crosses, we observed *P*. *ridibundus* chromosomes in 83 micronuclei (47%), 42 micronuclei (23%), 54 micronuclei (11%), 50 micronuclei (14%) and 72 micronuclei (21%), respectively (**Fig 4, S2 Table**). Thus, in L-E systems, the diploid hybrid tadpoles from the majority of crosses show high selectivity in eliminating *P*. *lessonae* chromosomes during gametogenesis. However, in tadpoles from other crosses, the elimination of *P*. *lessonae* and *P*. *ridibundus* chromosomes occurred with approximately equal likelihood, indicating either unselective elimination or elimination of different genomes by different germ cells.

In 16 diploid hybrid tadpoles from 2 different crosses of *P*. *lessonae* females and *P*. *esculentus* males from R-L-E systems, all tadpoles had germ cells; however, in 3 tadpoles, we observed low number of germ cells compared to *P*. *lessonae* tadpoles (**S2 Table)**. In the cytoplasm of germ cells from the gonads of 12 hybrid tadpoles, we found DAPI-positive micronuclei. Using 3D-FISH with the RrS1 probe, we investigated the gonads of 11 hybrid tadpoles from two crosses and found *P*. *ridibundus* chromosomes in 199 micronuclei (41%) and 49 micronuclei (13%), respectively (**Fig 4**). We conclude that tadpoles from some crosses of frogs from R-L-E systems are able to selectively eliminate the *P*. *lessonae* genome, while others exhibit an unselective pattern of genome elimination.

We also analysed the gonads of 26 hybrid tadpoles from 2 crosses of *P*. *ridibundus* females and *P*. *lessonae* males and one cross of a *P*. *lessonae* female and a *P*. *ridibundus* male. We found that in the first two crosses, 4 tadpoles lacked germ cells, while 10 tadpoles had an extremely low number of germ cells than were observed in *P*. *lessonae* tadpoles (**S2 Table)**. In a cross of a *P*. *lessonae* female and a *P*. *ridibundus* male, most tadpoles exhibited a normal number and morphology of germ cells within their gonads compared to what was observed in reciprocal crosses. In the gonads of 14 hybrid tadpoles, we observed DAPI-positive micronuclei. 3D-FISH was performed with an RrS1 repeat probe for the gonads of 6 hybrid tadpoles from two different crosses, and it revealed *P*. *ridibundus* chromosomes in 60 micronuclei (13%) and 13 micronuclei (14%), respectively (**Fig 4**). We conclude that although the majority of hybrid tadpoles produced from primary crosses have a low number of germ cells, some tadpoles exhibit a normal number and distribution of germ cells and can selectively eliminate the *P*. *lessonae* genome.

## Discussion

### Both aberrant germ cell development and selective genome elimination were observed during hybrid tadpoles gametogenesis

Interspecies hybridization and asexual reproduction require that hybrid offspring not only survive and develop but also instantly modify their gametogenesis to overcome sterility. In earlier studies, the effect of hybridization on progeny survival was shown to range from slightly increased levels of developmental abnormalities to almost complete mortality [9,13,29,42–44]. Herein, we focused on germ cell development during early gametogenesis in hybrid tadpoles obtained from primary and backcrosses. Our data demonstrated the absence or abnormal distribution of gonial cells in 54% of tadpoles from primary crosses, in 21% of tadpoles from crosses involving hybrid males from R-L-E systems and in 33% of tadpoles from crosses involving hybrid males from L-E systems. Previously, in R-E systems from eastern Ukraine, we observed tadpoles that did not have germ cells, indicating their potential sterility; however, the abundance of such tadpoles was extremely low [14]. It is still unknown whether tadpoles with abnormalities in germ cells development are able to develop beyond metamorphosis; however, high number of sterile males has been observed in the studied populations [25].

Significant sterility and gametogenetic aberrations in the studied tadpoles and adult frogs can be related to abnormal genome elimination, which occurs in gonial cells. Moreover, the presence of univalents in oocytes and spermatocytes as well as frequently observed aneuploid, di- and polyploid gametes in hybrids from different populations also indirectly indicates the absence of genome elimination and endoreplication in hybrids [13,14,30,45,46]. As genome elimination takes place premeiotically through micronuclei formation ([33,34], and our unpublished data), we focused on early stages of gonad development to evaluate success in genome elimination in hybrid tadpoles. In the current study we found micronuclei in the gonads of the majority of hybrid tadpoles from all types of crosses, indicating that the ability to eliminate genomes was present in the investigated hybrid tadpoles. However, incredibly low number of gonial cells exhibiting micronuclei formation was observed in some tadpoles, which indicates aberrant genome elimination and likely leads to their sterility. In early works, genome elimination has been previously shown for F1 hybrids [9,29] and hybrids from backcrosses [42]; however, genome elimination in the germ cells of tadpoles has not been directly observed thus far. Here, after FISH detection, we found that only a small portion of micronuclei include *P*. *ridibundus* chromosomes; thus, the majority of micronuclei included *P*. *lessonae* chromosomes in the majority of tadpoles from all types of crosses. Nevertheless, some tadpoles eliminate *P*. *ridibundus* and *P*. *lessonae* chromosomes almost equally. In the F1 hybrid, we found a high number of tadpoles with preferential elimination of *P*. *lessonae* chromosomes in micronuclei. The observed diploid *P*. *esculentus* from backcrosses and even primary crosses of parental species are able to selectively eliminate *P*. *lessonae* chromosomes, indicating the emergence of hybridogenetic hybrids in all studied population systems. Based on our data, we propose that a high number of adult hybrid frogs with aberrant gonads, low level or absence of functional gametes [25 and this study] as well as a high apoptotic rate of gonial cells in tadpoles caused by unselective genome elimination during early development. Only those germ cells that successfully eliminated only *P*. *lessonae* chromosomes can divide further and produce gametes.

### Hints at evolution in studied hybrid complexes

**Fig 5:**
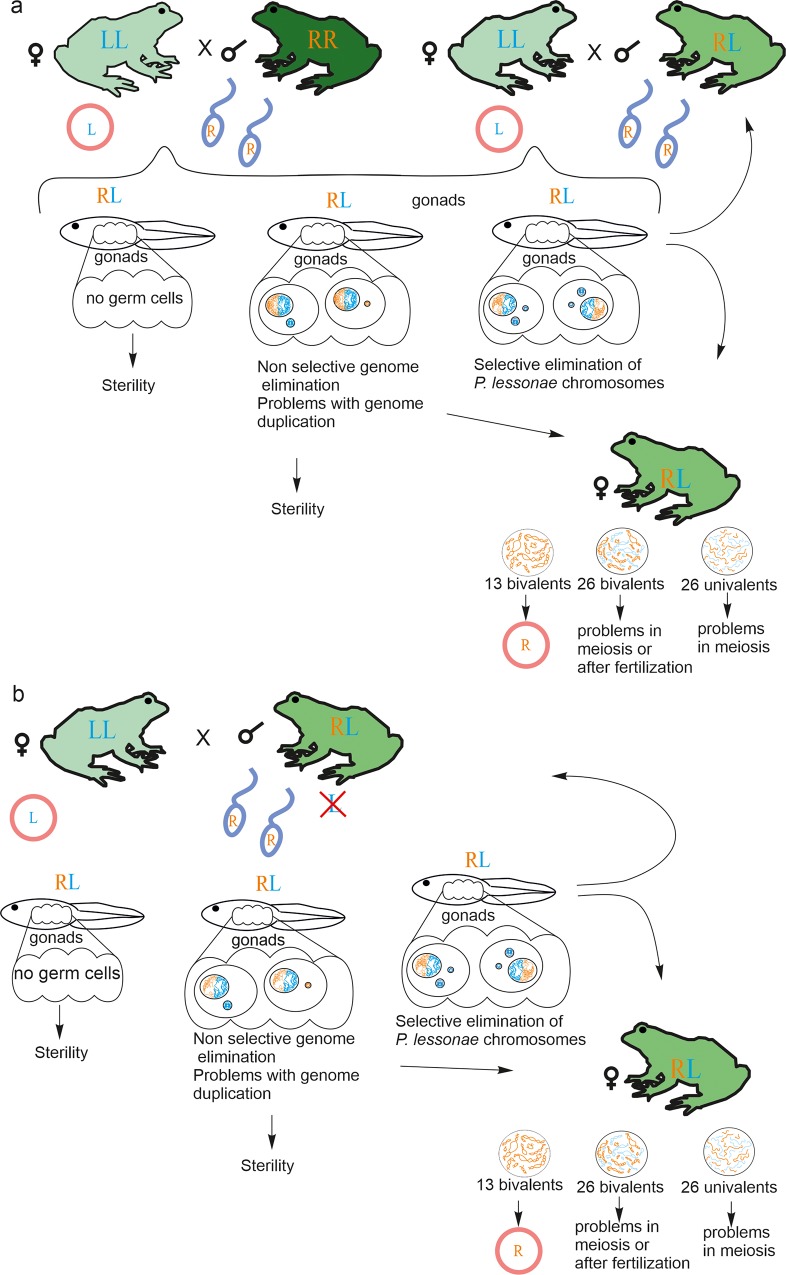
**Schematic overview of hybrid frog reproduction in the studied R-L-E (a) and L-E (b) population systems of the *Pelophylax esculentus* complex.** Scheme includes reproductive interactions among studied genotypes and resulting progeny that have varying ability to eliminate one of the parental genomes during gametogenesis and thus varying ability to produce gametes.

Asexual hybrids can influence speciation in several ways. Asexual hybrids can not only restrict gene flow between parental species because recombination is prevented between their genomes but also mediate speciation themselves [18–20,47]. In this case, hybrid gametogenesis should first be modified to overcome sterility and then changed back again to return to sexual reproduction [12,48–50]. Together with previous findings, we observed the ability of at least some F1 hybrids to perform *P*. *lessonae* genome elimination, revealing a crucial step in establishing hybridogenetic reproduction. We suggest that initially, studied R-L-E systems can emerge after contact of *P*. *ridibundus* and *P*. *lessonae* (**Fig 5**). However, in such populations, hybrids usually produce gametes with *P*. *ridibundus* genome allowing them to reproduce only when they cross with *P*. *lessonae* individuals. Thus, here we confirm two different mechanisms of hybrid appearance in R-L-E systems: from primary crosses of parental species and from crosses of hybrid individuals with *P*. *lessonae*.

Our results partially support the hypothesis of an independent origin of hybridogenetic hybrids in studied population systems. However, the distribution of *P*. *esculentus* is extremely broad and almost ubiquitously coincides with the range of parental species. Nevertheless, in some localities, crosses of *P*. *ridibundus* with *P*. *lessonae* do not always lead to the appearance of viable and fertile *P*. *esculentus*. This can be explained by high levels of polymorphisms that have been previously described between different populations of *P*. *ridibundus* and *P*. *lessonae* [15,16,51]. Moreover, introgression of alleles from western *P*. *ridibundus* related form *P*. cf. *bedriagae* can also affect the possibility of genome elimination [22]. Such introgressions may also affect the formation of asexual hybrids, as in studied area specific alleles were quite frequent among *P*. *ridibundus* but rarely observed in hybrids [22]. This is in accordance with the fact that only “pure” *P*. *ridibundus* can induce hybridogenesis after crosses with *P*. *lessonae* or several *lessonae*-related species, while even the closely related Balcan taxon *P*. *kurtmuelleri* seems unable to produce asexual hybrids [52].

Taking into account the similarity in mechanisms of maintaining L-E and R-L-E systems in the Mari El Republic, we suggest their common origin in the studied hybrid complexes. Hybridogenetic *P*. *esculentus* can either displace parental *P*. *ridibundus* or invade habitats that are suitable for *P*. *lessonae*, leading to the appearance of L-E systems. We found that hybrid males from both L-E and R-L-E systems produced hybrid tadpoles via the formation of gametes with the *P*. *ridibundus* genome (**Fig 5**). Our results are in close agreement with earlier data about sperm genome composition in *P*. *esculentus* males from studied locations [25,26]. The formation of sperm with the *P*. *ridibundus* genome by diploid hybrid males was widely observed in different L-E systems located in western Russia, Poland, Germany, Slovakia, Latvia, and Belarus [17,28,32,47,51,53]. Nevertheless, in contrast to previously described L-E systems, hybrid females in the studied systems possessed many irregularities in genome elimination and duplication, leading to the predominant formation of oocytes with univalents. Oocytes with univalents likely cannot progress beyond meiosis. On the other hand, the occurrence of *P*. *ridibundus* mtDNA in hybrid females (11%) in some L-E systems indicates that individual hybrid females are able to reproduce [26]. Nevertheless, hybrid reproduction in the Mari El population systems depends primarily on hybrid males producing *P*. *ridibundus* gametes.

The achievement of sexual reproduction via the formation of polyploid animals, in particular triploids, which are also known as the “triploid bridge” is an important stage in the hybrid speciation concept [2, 50, 54, 55]. Although triploid animals were not detected in the Mari El population systems, we observed the ability of diploid hybrid females to form oocytes with doubled chromosomal sets, which is a prerequisite for the emergence of triploids in different hybrid frog populations [13,14,28,56–58].

## Conclusions

This study demonstrates a notable link between cytological mechanisms causing asexuality and their impact on hybrid populations and speciation. We found that asexual reproduction is not always achievable for hybrid animals, since some tadpoles exhibit abnormal development of germ cells and eliminate chromosomes of both parental species with variable rates of selectivity. However, even in F1 crosses, some hybrid tadpoles form micronuclei that preferentially include *P*. *ridibundus* chromosomes; thus, such tadpoles are able to eliminate the genome selectively.

Based on our results, we propose a model of hybrid frog reproduction in the studied R-L-E and L-E systems **(Fig 5)**. We found that in R-L-E systems, hybridogenetic *P*. *esculentus* can emerge from crosses of parental species and from crosses of *P*. *esculentus* males with *P*. *lessonae* females. Surprisingly, hybrid reproduction in both R-L-E and L-E systems depends basically on *P*. *esculentus* males producing gametes with *P*. *ridibundus* genome but not on *P*. *esculentus* females. Hybrid females in the investigated systems exhibit numerous abnormalities in genome elimination and/or endoreplication, leading to prevalent formation of oocytes with 26 univalents. However, some hybrid females produced oocytes with 13 bivalents of *P*. *ridibundus* as well as rare oocytes with 26 bivalents, which are subsequently able to give rise to triploid hybrids. Thus, populations from the western border of *P*. *esculentus* range in the Mari El Republic represent an example of the initial stages of speciation via interspecies hybridization.

## Supporting information

S1 Table List of European water frogs from R-L-E and L-E systems in the study. Crosses of parental individuals are marked in orange, crosses between *P*. *lessonae* individuals are marked in red, crosses of *P*. *lessonae* with diploid hybrids from L-E systems are marked in blue and those with R-L-E systems are marked in green. Frogs used for lampbrush chromosome analysis from L-E systems (marked blue) and R-L-E systems (marked green).
(PDF)

S2 Table Analysis of gonads dissected from tadpoles with identification of genome in the micronuclei. Tadpoles obtained from crosses of parental individuals (marked orange), two *P*. *lessonae* individuals (marked red) and *P*. *lessonae* individuals and hybrids from L-E systems (marked blue) and R-L-E systems (marked green).
(PDF)

S1 Fig Identification of *P*. *ridibundus* lampbrush chromosomes using FISH for the centromeric RrS1 repeat. Lampbrush chromosome sets, including 26 univalents (a) and 26 bivalents (b), among which 13 uni- or bivalents have a signal in the centromeric region, thus corresponding to *P*. *ridibundus* chromosomes; another 13 uni- or bivalents do not have a signal in the centromeric region, thus corresponding to *P*. *lessonae* chromosomes. The chromosomal set represented in **S1A Fig** corresponds to **Fig 3B**. The chromosomal set represented in **S1B Fig** corresponds to **Fig 3C**.
(PDF)
