# Supplementary material for: Variation in hybridogenetic hybrid emergence between populations of water frogs from the Pelophylax esculentus complex
Source: PLoS One. 2019 Nov 1;14(11):e0224759. doi: 10.1371/journal.pone.0224759 (PMC6824575; doi:10.1371/journal.pone.0224759)
Supplement: S1 Table — Crosses of parental individuals are marked in orange, crosses between P. lessonae individuals are marked in red, crosses of P. lessonae with diploid hybrids from L-E systems are marked in blue and those with R-L-E systems are marked in green. Frogs used for lampbrush chromosome analysis from L-E systems (marked blue) and R-L-E systems (marked green). (PDF) [file pone.0224759.s001.pdf]

S1 Table 1

Succsessful crosses							
№	Frog`s species	Genome size	Sex	Methods of frogs identification	Frogs id	Locality	Coordinates
1	*P. ridibundus*	16,352	f	DNA flow cytometry	6_2015_f	Chermyshevo	56.196386 N, 46.519268 E
2	*P. lessonae*	13,930	m_1	DNA flow cytometry	6_2015_m1	Chermyshevo	56.196386 N, 46.519268 E
3	*P. lessonae*	13,905	m_2	DNA flow cytometry	6_2015_m2	Chermyshevo	56.196386 N, 46.519268 E
4	*P. ridibundus*	16,123	f	DNA flow cytometry	16_2015_f	Medvedevo	56.641235 N, 47.752490 E
5	*P. lessonae*		m	Karyotyping with FISH	16_2015_m	Krasnooktyabr'skiy	56.681029 N, 47.678995 E
6	*P. ridibundus*		f	Karyotyping with FISH	11_2016_f	Medvedevo	56.641235 N, 47.752490 E
7	*P. lessonae*		m	Karyotyping with FISH	11_2016_m1	Kuguvan	56.786141 N, 47.784421 E
8	*P. lessonae*		m	Karyotyping with FISH	11_2016_m2	Krasnooktyabr'skiy	56.681029 N, 47.678995 E
9	*P. ridibundus*		f	Karyotyping with FISH	13_2016_f1	Medvedevo	56.641235 N, 47.752490 E
10	*P. lessonae*		m	Karyotyping with FISH	13_2016_m1	Kuguvan	56.786141 N, 47.784421 E
11	*P. lessonae*		m	Karyotyping with FISH	13_2016_m2	Kuguvan	56.786141 N, 47.784421 E
12	*P. lessonae*		f	Karyotyping with FISH	1_2017_f	Pyn'zhedur	56.613111 N, 47.931808 E
13	*P. ridibundus*		m	Karyotyping with FISH	1_2017_m	Sosnovaya Roszha, Yoshkar-Ola	56.281948 N, 46.898717 E
14	*P. lessonae*		f	Karyotyping with FISH	11_2015_f	Kuguvan	56.786141 N, 47.784421 E
15	*P. esculentus*	14,968	m	DNA flow cytometry	11_2015_m	Kuguvan	56.786141 N, 47.784421 E
16	*P. lessonae*	13,801	f	DNA flow cytometry	12_2015_f	Kuguvan	56.786141 N, 47.784421 E
17	*P. esculentus*	14,969	m	DNA flow cytometry	12_2015_m	Kuguvan	56.786141 N, 47.784421 E
18	*P. lessonae*		f	Karyotyping with FISH	13_2015_f	Kuguvan	56.786141 N, 47.784421 E
19	*P. esculentus*	14,975	m	DNA flow cytometry	13_2015_m	Kuguvan	56.786141 N, 47.784421 E
20	*P. lessonae*	13,758	f	DNA flow cytometry	14_2015_f	Kuguvan	56.786141 N, 47.784421 E
21	*P. esculentus*	14,986	m	DNA flow cytometry	14_2015_m	Kuguvan	56.786141 N, 47.784421 E
22	*P. lessonae*	13,700	f	DNA flow cytometry	26_2015_f	Shusher	56.672423 N, 47.261847 E
23	*P. esculentus*	14,948	m	DNA flow cytometry	26_2015_m	Kuguvan	56.786141 N, 47.784421 E
24	*P. lessonae*	13,883	f	DNA flow cytometry	27_2015_f	Kuguvan	56.786141 N, 47.784421 E
25	*P. esculentus*	14,959	m	DNA flow cytometry	27_2015_m	Kuguvan	56.786141 N, 47.784421 E
26	*P. lessonae*		f	Karyotyping with FISH	5_2016_f	Kuguvan	56.786141 N, 47.784421 E

27	*P. esculentus*		m	Karyotyping with FISH	5_2016_m	Kuguvan	56.786141 N, 47.784421 E
28	*P. lessonae*		f	Karyotyping with FISH	6_2016_f	Krasnooktyabr'skiy	56.681029 N, 47.678995 E
29	*P. esculentus*		m	Karyotyping with FISH	6_2016_m	Kuguvan	56.786141 N, 47.784421 E
30	*P. lessonae*	13,795	f	DNA flow cytometry	19_2015_f	Shusher	56.672423 N, 47.261847 E
31	*P. esculentus*	15,137	m	DNA flow cytometry	19_2015_m	Krasnooktyabr'skiy	56.681029 N, 47.678995 E
32	*P. lessonae*	13,785	f_1	DNA flow cytometry	30_2015_f1	Chermyshevo	56.196386 N, 46.519268 E
33	*P. lessonae*	13,811	f_2	DNA flow cytometry	30_2015_f2	Chermyshevo	56.196386 N, 46.519268 E
34	*P. esculentus*	15,035	m	DNA flow cytometry	30_2015_m	Chermyshevo	56.196386 N, 46.519268 E
35	*P. lessonae*		f	Morphological examination	2_2017_f	Pyn'zhedur	56.613111 N, 47.931808 E
36	*P. lessonae*		m	Morphological examination	2_2017_m	Pyn'zhedur	56.613111 N, 47.931808 E

Unseccessful crosses							
№	Frog`s species	Genome size	Sex	methods of frogs identification	Frogs id	Locality	Coordinates
1	*P. lessonae*	13,916	f	DNA flow cytometry	10_2015_f	Shusher	56.672423 N, 47.261847 E
2	*P. ridibundus*	16,368	m	DNA flow cytometry	10_2015_m	Medvedevo	56.641235 N, 47.752490 E
3	*P. ridibundus*		f	Karyotyping with FISH	17_2015_f	Medvedevo	56.641235 N, 47.752490 E
4	*P. lessonae*	13,791	m	DNA flow cytometry	17_2015_m	Krasnooktyabr'skiy	56.681029 N, 47.678995 E
5	*P. ridibundus*	16,116	f	DNA flow cytometry	28_2015_f	Medvedevo	56.641235 N, 47.752490 E
6	*P. lessonae*	13,682	m	DNA flow cytometry	28_2015_m	Kuguvan	56.786141 N, 47.784421 E
7	*P. esculentus*	15,178	f	DNA flow cytometry	1_2015_f	Kuguvan	56.786141 N, 47.784421 E
8	*P. lessonae*	14,045	m	DNA flow cytometry	1_2015_m	Kuguvan	56.786141 N, 47.784421 E
9	*P. esculentus*	15,145	f	DNA flow cytometry	2_2015_f	Kuguvan	56.786141 N, 47.784421 E
10	*P. lessonae*	13,984	m	DNA flow cytometry	2_2015_m	Kuguvan	56.786141 N, 47.784421 E
11	*P. esculentus*	15,167	f	DNA flow cytometry	3_2015_f	Kuguvan	56.786141 N, 47.784421 E
12	*P. lessonae*	13,927	m	DNA flow cytometry	3_2015_m	Kuguvan	56.786141 N, 47.784421 E
13	*P. esculentus*	15,103	f	DNA flow cytometry	4_2015_f	Kuguvan	56.786141 N, 47.784421 E
14	*P. lessonae*		m	Karyotyping with FISH	4_2015_m	Kuguvan	56.786141 N, 47.784421 E
15	*P. esculentus*	14,966	f	DNA flow cytometry	7_2015_f	Oshlamoochash	56.812210 N, 47.760231 E
16	*P. lessonae*	13,627	m	DNA flow cytometry	7_2015_m	Krasnooktyabr'skiy	56.681029 N, 47.678995 E
17	*P. esculentus*	14,876	f	DNA flow cytometry	22_2015_f	Krasnooktyabr'skiy	56.681029 N, 47.678995 E

18	*P. esculentus*	14,927	m	DNA flow cytometry	22_2015_m	Shusher	56.672423 N, 47.261847 E
19	*P. lessonae*	13,668	f_1	DNA flow cytometry	24_2015_f1	Krasnooktyabr'skiy	56.681029 N, 47.678995 E
20	*P. lessonae*	13,673	f_2	DNA flow cytometry	24_2015_f2	Krasnooktyabr'skiy	56.681029 N, 47.678995 E
21	*P. esculentus*	14,913	m	DNA flow cytometry	24_2015_m1	Shusher	56.672423 N, 47.261847 E
22	*P. lessonae*	13,684	f	DNA flow cytometry	31_2015_f1	Kuguvan	56.786141 N, 47.784421 E
23	*P. lessonae*	13,724	f	DNA flow cytometry	31_2015_f2	Kuguvan	56.786141 N, 47.784421 E
24	*P. esculentus*	14,805	m	DNA flow cytometry	31_2015_m1	Oshlamoochash	56.812210 N, 47.760231 E
25	*P. lessonae*	13,705	f	DNA flow cytometry	32_2015_f	Kuguvan	56.786141 N, 47.784421 E
26	*P. esculentus*	14,869	m	DNA flow cytometry	32_2015_m	Oshlamoochash	56.812210 N, 47.760231 E
27	*P. lessonae*	13,714	f	DNA flow cytometry	34_2015_f	Chermyshevo	56.196386 N, 46.519268 E
28	*P. esculentus*	15,001	m	DNA flow cytometry	34_2015_m	Kuguvan	56.786141 N, 47.784421 E
29	*P. ridibundus*	16,079	f	DNA flow cytometry	37_2015_f	Chermyshevo	56.196386 N, 46.519268 E
30	*P. esculentus*	14,922	m	DNA flow cytometry	37_2015_m	Kuguvan	56.786141 N, 47.784421 E
31	*P. lessonae*	13,767	f	DNA flow cytometry	15_2016_f	Kuguvan	56.786141 N, 47.784421 E
32	*P. esculentus*	15,065	m	DNA flow cytometry	15_2016_m	Kuguvan	56.786141 N, 47.784421 E
33	*P. esculentus*	15,129	f	DNA flow cytometry	5_2015_f	Chermyshevo	56.196386 N, 46.519268 E
34	*P. ridibundus*	16,312	m	DNA flow cytometry	5_2015_m	Oshlamoochash	56.812210 N, 47.760231 E
35	*P. esculentus*	15,169	f	DNA flow cytometry	8_2015_f	Medvedevo	56.641235 N, 47.752490 E
36	*P. lessonae*	14,013	m	DNA flow cytometry	8_2015_m	Medvedevo	56.641235 N, 47.752490 E
37	*P. esculentus*	14,922	f	DNA flow cytometry	9_2015_f	Krasnooktyabr'skiy	56.681029 N, 47.678995 E
38	*P. lessonae*	13,817	m	DNA flow cytometry	9_2015_m	Krasnooktyabr'skiy	56.681029 N, 47.678995 E
39	*P. esculentus*	14,922	f	DNA flow cytometry	23_2015_f	Krasnooktyabr'skiy	56.681029 N, 47.678995 E
40	*P. lessonae*	13,726	m	DNA flow cytometry	23_2015_m	Kuguvan	56.786141 N, 47.784421 E
41	*P. lessonae*	13,801	f	DNA flow cytometry	25_2015_f	Chermyshevo	56.196386 N, 46.519268 E
42	*P. esculentus*	14,987	m	DNA flow cytometry	25_2015_m	Chermyshevo	56.196386 N, 46.519268 E
43	*P. ridibundus*	15,981	f	DNA flow cytometry	29_2015_f	Medvedevo	56.641235 N, 47.752490 E
44	*P. esculentus*	14,922	m	DNA flow cytometry	29_2015_m	Krasnooktyabr'skiy	56.681029 N, 47.678995 E
45	*P. ridibundus*		f	DNA flow cytometry	33_2015_f	Medvedevo	56.641235 N, 47.752490 E
46	*P. esculentus*	14,975	m	DNA flow cytometry	33_2015_m	Medvedevo	56.641235 N, 47.752490 E

Frogs additionally used for lampbrush chromosome analysis

№	Frog`s species	Genome size	Sex	methods of frogs identificatio	Frogs id	Locality	Coordinates
1	*P. esculentus*		f	Karyotyping with FISH	1K_2017_f	Kuguvan	56.786141 N, 47.784421 E
2	*P. esculentus*		f	Karyotyping with FISH	2K_2017_f	Kuguvan	56.786141 N, 47.784421 E
3	*P. esculentus*		f	Karyotyping with FISH	3K_2017_f	Kuguvan	56.786141 N, 47.784421 E
4	*P. esculentus*		f	Karyotyping with FISH	4K_2017_f	Kuguvan	56.786141 N, 47.784421 E
5	*P. esculentus*		f	Karyotyping with FISH	5K_2017_f	Kuguvan	56.786141 N, 47.784421 E
6	*P. esculentus*		f	Karyotyping with FISH	1N_2017_f	Nol'ka	56.584767 N, 47.933461 E
7	*P. esculentus*		f	Karyotyping with FISH	1M_2017_f	Medvedevo	56.641235 N, 47.752490 E
8	*P. esculentus*		f	Karyotyping with FISH	5_2014_f	Chermyshevo	56.196386 N, 46.519268 E
9	*P. esculentus*		f	Karyotyping with FISH	2_2014_f	Chermyshevo	56.196386 N, 46.519268 E

Population system
R-L-E system
R-L-E system
R-L-E system
R-L-E system
R-L-E system
R-L-E system
L-E system
R-L-E system
R-L-E system
L-E system
L-E system
L system
R system
L-E system
L-E system
L-E system
L-E system
L-E system
L-E system
L-E system
L-E system
L-E system
L-E system
L-E system
L-E system
L-E system

L-E system
R-L-E system
L-E system
L-E system
R-L-E system
R-L-E system
R-L-E system
R-L-E system
L system
L system

Population system
L-E system
R-L-E system
R-L-E system
R-L-E system
R-L-E system
L-E system
L-E system
L-E system
L-E system
L-E system
L-E system
L-E system
L-E system
L-E system
L-E system
R-L-E system
R-L-E system

L-E system
R-L-E system
R-L-E system
L-E system
L-E system
L-E system
L-E system
L-E system
L-E system
R-L-E system
L-E system
R-L-E system
L-E system
L-E system
L-E system
R-L-E system
L-E system
R-L-E system
R-L-E system
R-L-E system
R-L-E system
R-L-E system
L-E system
R-L-E system
R-L-E system
R-L-E system
R-L-E system
R-L-E system
R-L-E system

Population system
L-E system
L-E system
L-E system
L-E system
L-E system
R-L-E system
R-L-E system
R-L-E system
R-L-E system
