# Supplementary material for: Variation in hybridogenetic hybrid emergence between populations of water frogs from the Pelophylax esculentus complex
Source: PLoS One. 2019 Nov 1;14(11):e0224759. doi: 10.1371/journal.pone.0224759 (PMC6824575; doi:10.1371/journal.pone.0224759)
Supplement: S2 Table — Tadpoles obtained from crosses of parental individuals (marked orange), two P. lessonae individuals (marked red) and P. lessonae individuals and hybrids from L-E systems (marked blue) and R-L-E systems (marked green). (PDF) [file pone.0224759.s002.pdf]

S2 Table 2

Crosses type	Number of analyzed tadpoles	Crosses id	Tadpoles ploidy	Gosner stage	Number of germ cells	Number of cells with micronuclei	Number of micronuclei	Number of micronuclei with signals
female RR x male LL	1	6_2015	2n	28	2	no		
	2	6_2015	2n	34	392	53		
	3	6_2015	2n	32	255	63		
	4	6_2015	2n	31	18	no		
	5	6_2015	2n	31	43	2		
	6	6_2015	2n	33	199	37		
	7	6_2015	2n	30	1	no		
	8	6_2015	2n	30	7	no		
	9	6_2015	2n	29	no	no		
	10	6_2015	2n	31	23	1		
	11	6_2015	2n	28	1	no		
	12	6_2015	2n	29	1	no		
	13	6_2015	2n	30	no	no		
	14	6_2015	2n	30	3	no		
	15	6_2015	2n	32	228	56	81	13
female LL x male RR	1	1_2017	2n	34	744	165	215	27
	2	1_2017	2n	32	307	78	100	3
	3	1_2017	2n	29	71	27	37	11
	4	1_2017	2n	30	137	24	29	3
	5	1_2017	2n	34	607	91	111	16
female RR x male LL	1	16_2015	2n	28	no	no		
	2	16_2015	2n	33	178	55		
	3	16_2015	2n	30	1	no		
	4	16_2015	2n	29	21	1		
	5	16_2015	2n	30	no	no		
	6	16_2015	2n	31	21	1		
female LL x male RL	1	11_2015	2n	32	11	1		
	2	11_2015	2n	30	27	no		
	3	11_2015	2n	31	40	no		
	4	11_2015	2n	32	165	66	93	50

	5	11_2015	2n	33	52	20	24	9
	6	11_2015	2n	32	68	41	61	24
female LL x male RL	1	12_2015	2n	31	20	2		
	2	12_2015	2n	33	160	15		
	3	12_2015	2n	30	24	7		
	4	12_2015	2n	34	115	58		
	5	12_2015	2n	34	176	69		
	6	12_2015	2n	35	198	40	45	14
	7	12_2015	2n	34	252	89	121	23
	8	12_2015	2n	32	65	15	21	5
female LL x male RL	1	13_2015	2n	32	5	2		
	2	13_2015	2n	32	7	no		
	3	13_2015	2n	33	413	120	150	26
	4	13_2015	2n	34	503	194	267	24
	5	13_2015	2n	30	125	40	56	4
female LL x male RL	1	14_2015	2n	34	377	96		
	2	14_2015	2n	33	97	38		
	3	14_2015	2n	34	249	76		
	4	14_2015	2n	32	161	22		
	5	14_2015	2n	31	no			
	6	14_2015	2n	33	12	2		
	7	14_2015	2n	32	111	11		
	8	14_2015	2n	32	no			
	9	14_2015	2n	33	305	74		
	10	14_2015	2n	34	289	38		
	11	14_2015	2n	30	126	53		
	12	14_2015	2n	35	568	108	152	27
	13	14_2015	2n	34	358	139	205	23
female LL x male RL	1	26_2015	2n	34	428	127	173	41
	2	26_2015	2n	34	572	79	96	14
	3	26_2015	2n	32	171	14	21	6
	4	26_2015	2n	33	217	49	60	11
female LL x male RL	1	27_2015	2n	32	73	34		

	2	27_2015	2n	29	25	9		
	3	27_2015	2n	28	23	12		
	4	27_2015	2n	31	55	21		
	5	27_2015	2n	33	109	13		
female LL x male RL	1	19_2015	2n	29	50	23		
	2	19_2015	2n	30	115	6		
	3	19_2015	2n	34	359	34		
	4	19_2015	2n	32	14	no		
	5	19_2015	2n	32	76	11	13	7
	6	19_2015	2n	35	244	106	174	75
	7	19_2015	2n	34	137	37	57	22
	8	19_2015	2n	29	73	18	26	8
	9	19_2015	2n	30	81	26	45	13
	10	19_2015	2n	33	239	88	133	51
	11	19_2015	2n	31	61	28	42	23
female LL x male RL	1	30_2015	2n	35	526	171	233	25
	2	30_2015	2n	35	207	22	28	7
	3	30_2015	2n	34	342	86	111	17
	4	30_2015	2n	32	27	3	5	no
	5	30_2015	2n	30	15			
Female LL x male LL	1	2_2017	2n	34	245			
	2	2_2017	2n	32	118			
	3	2_2017	2n	34	184			
