# Supplementary figures and images for: Variation in hybridogenetic hybrid emergence between populations of water frogs from the Pelophylax esculentus complex
Source: PLoS One. 2019 Nov 1;14(11):e0224759. doi: 10.1371/journal.pone.0224759 (PMC6824575; doi:10.1371/journal.pone.0224759)

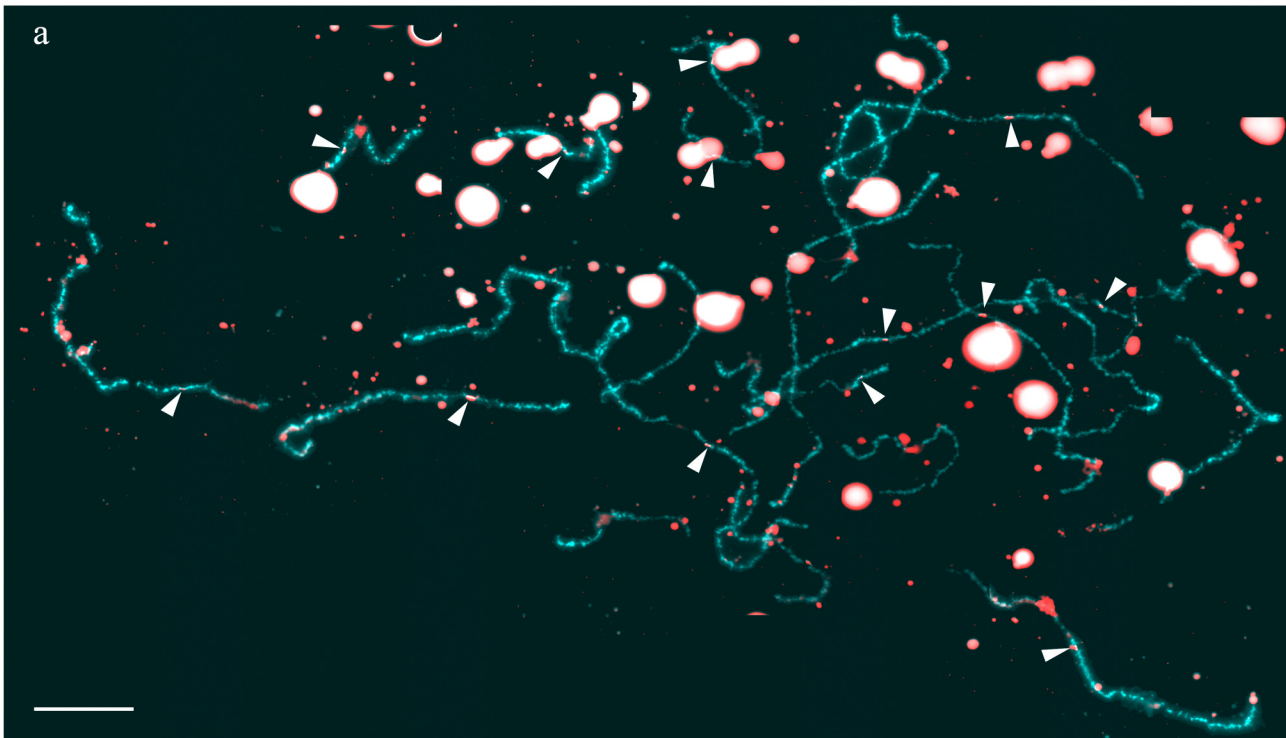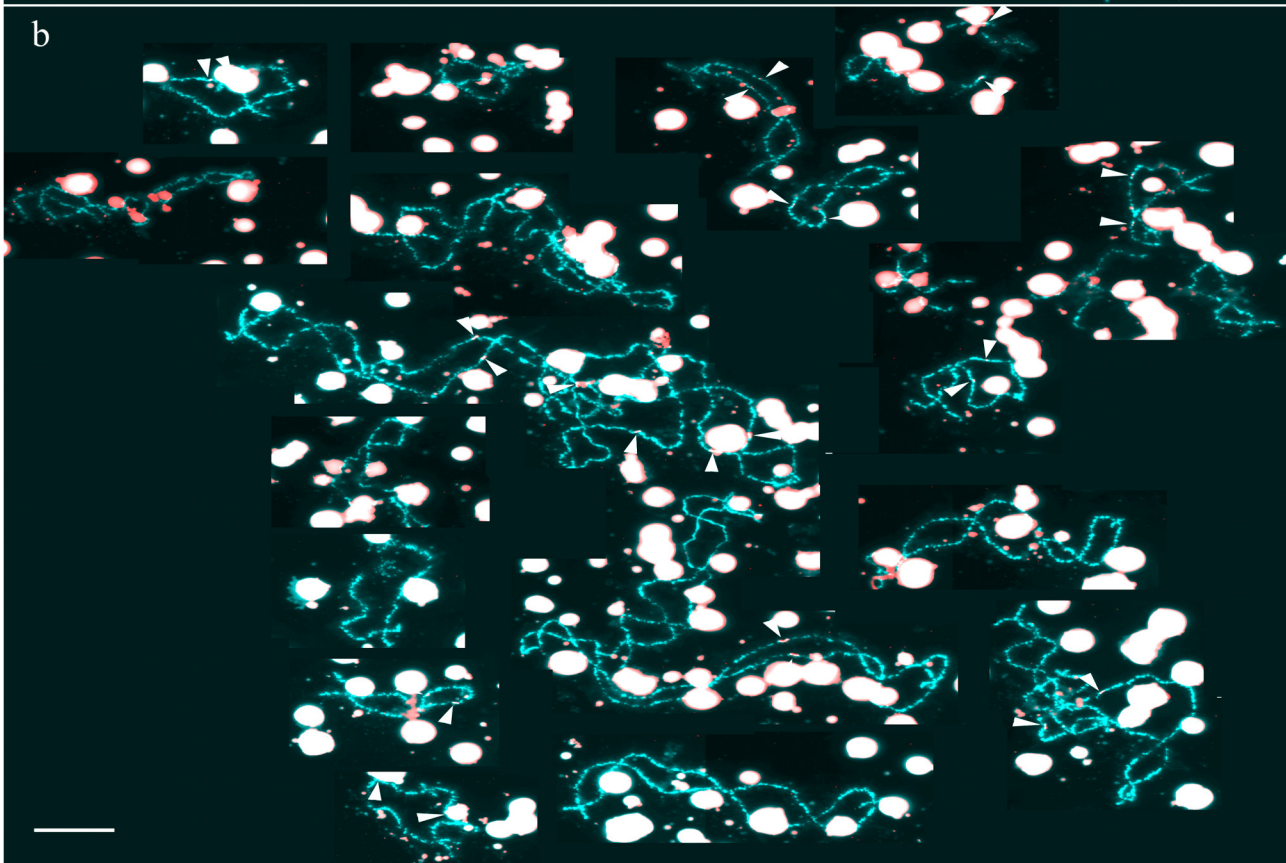

Supplement: S1 Fig — Lampbrush chromosome sets, including 26 univalents (a) and 26 bivalents (b), among which 13 uni- or bivalents have a signal in the centromeric region, thus corresponding to P. ridibundus chromosomes; another 13 uni- or bivalents do not have a signal in the centromeric region, thus corresponding to P. lessonae chromosomes. The chromosomal set represented in S1A Fig corresponds to Fig 3B. The chromosomal set represented in S1B Fig corresponds to Fig 3C. (PDF) [file pone.0224759.s003.pdf]
